# Psychometric properties of the short version of the Foreign Language Learning Enjoyment Scale in the Classroom (EDALEC) in Peruvian youth

**DOI:** 10.3389/fpsyg.2026.1798560

**Published:** 2026-09-11

**Authors:** Hadassy Yolanda Franco-Girón, Nancy Mirian Quinto-Machaca, Almendra Jazmin Chapoñan-Chaname, Joel Palomino-Ccasa, Segundo Salatiel Malca-Peralta

**Affiliations:** 1 Escuela de Educación, Universidad Peruana Unión, Lima, Peru; 2 Escuela de Psicología, Universidad Peruana Unión, Lima, Peru

**Keywords:** affective behavior, foreign language learning, measurement invariance, psychometrics, student attitudes, test validity, young adults

## Abstract

**Introduction:**

Enjoyment in foreign language learning has become an important construct in educational research and second language acquisition; however, psychometric evidence for instruments assessing this construct in Latin American contexts remains limited. This study aimed to examine the psychometric properties of the short version of the Foreign Language Enjoyment Scale in the Classroom (EDALEC) in Peruvian youth.

**Methods:**

Using an instrumental design, 967 participants of both sexes were recruited and randomly divided into two independent subsamples to conduct exploratory and confirmatory factor analyses. The psychometric properties of the instrument were evaluated through factor analyses, measurement invariance across sex, convergent and discriminant validity, and reliability analyses.

**Results:**

The exploratory factor analysis, supported by parallel analysis, identified a three-factor structure comprising Teacher-induced enjoyment, Personal enjoyment, and Social enjoyment. The confirmatory factor analysis supported this structure and demonstrated excellent model fit (*χ*^2^ = 48, df = 24, CFI = 0.999, TLI = 0.998, RMSEA = 0.040, SRMR = 0.008). The instrument also demonstrated configural, metric, scalar, and strict measurement invariance across sex. Convergent validity was supported by Average Variance Extracted values above the recommended threshold, whereas discriminant validity was confirmed through heterotrait–monotrait ratio values below .85. Reliability estimates ranged from *α* = 0.77–0.92 and *ω* = 0.75–0.88, indicating adequate internal consistency across the three dimensions.

**Discussion:**

The findings provide evidence of structural validity, measurement invariance, and internal consistency for the short version of the EDALEC in Peruvian youth. This instrument may be useful for assessing foreign language enjoyment in Latin American educational contexts.

## 1 Introduction

In recent years, the study of emotions in foreign language (FL) learning has gained increasing attention, as empirical evidence has demonstrated their significant impact on pedagogical effectiveness and academic achievement (Wang and Jiang, 2022). Educational environments have traditionally been dominated by quantitative assessments, grades, and standardized metrics; however, sociocultural and psycho-emotional factors have been shown to play a crucial role in teaching and learning processes (Derakhshan and Fathi, 2024). Within this framework, Dewaele and MacIntyre (2014) introduced the construct of Foreign Language Enjoyment (FLE), defined as a positive emotion that emerges when learners’ psychological needs are satisfied in the classroom. This emotion has been consistently associated with meaningful learning outcomes, whereas negative emotions such as foreign language anxiety tend to inhibit the learning process.

To assess FLE, Dewaele and MacIntyre (2014) developed a 21-item scale based on the Interest and Enjoyment subscale proposed by Ryan et al. (1990), which was later adapted into Spanish under the name Escala de Disfrute con el Aprendizaje de la Lengua Extranjera en el Aula (EDALEC). The original unidimensional version, using a five-point Likert-type response format, demonstrated strong psychometric properties, with internal consistency coefficients of *α* = 0.86 in the original version and *α* = 0.91 in the Spanish adaptation. Additionally, moderate divergent validity with measures of classroom anxiety has been reported (Acosta-Manzano and Barrios, 2022).

Despite these strengths, the length of the original scale has been questioned in applied research contexts due to its potential effects on participant fatigue and response rates (Botes et al., 2021; Acosta-Manzano and Barrios, 2022). This concern has motivated the development of abbreviated versions that retain the psychometric robustness of the original instrument while enhancing its practicality. Notable examples include the 10-item version proposed by Dewaele et al. (2018), comprising two dimensions, Social enjoyment and private enjoyment (*α* = 0.86); the 11-item version by Li et al. (2018), which identified three dimensions, private enjoyment, teacher-related enjoyment, and classroom atmosphere (*α* = 0.88); and the 16-item version developed by Jin and Zhang (2019), which organized enjoyment around English learning, teacher support, and peer support, demonstrating superior validity compared to previous versions. More recently, Botes et al. (2021) developed a 9-item short form structured around three higher-order factors, Teacher-induced enjoyment (*α* = 0.92), Personal enjoyment (*α* = 0.71), and Social enjoyment (*α* = 0.77). When adapted into Spanish, this version exhibited high internal consistency (*α* = 0.85) and acceptable convergent and divergent validity (Wang and Jiang, 2022).

Despite these advances, the scientific literature reveals a significant gap in the validation of Foreign Language Enjoyment Scales in Latin American contexts. To date, only one psychometric validation of the Spanish EDALEC has been reported, conducted in Spain with a relatively small European sample (*n* = 184) (Acosta-Manzano and Barrios, 2022). No validation studies have been conducted with Spanish-speaking populations in Latin America, representing a critical gap in psychometric research on foreign language enjoyment in this region. This lack of evidence limits the applicability of the scale in Latin American educational settings, where cultural and linguistic particularities may influence students’ responses and, consequently, the measurement of foreign language enjoyment.

Having short-form psychometric instruments that preserve robust measurement properties is particularly important for applied educational research, as they reduce respondent burden, minimize fatigue, and optimize data collection in time-constrained educational contexts. At the same time, brief scales tend to improve response rates without compromising validity or reliability (Jebb et al., 2021). Therefore, the psychometric validation of the short version of the EDALEC in Latin American contexts is essential not only to ensure its cultural and linguistic relevance but also to provide evidence of internal consistency, construct validity, and measurement reliability.

In response to this need, the aim of the present study was to analyze the psychometric properties of the 9-item short version of the EDALEC in a representative sample of Peruvian youth. By doing so, this study seeks to contribute to the availability of psychometrically supported, culturally adapted, and efficient instruments for both research and educational practice within the Latin American context.

## 2 Methods

### 2.1 Design

This study employed a cross-sectional instrumental design aimed at evaluating the psychometric properties of measurement scales (Ato et al., 2013).

### 2.2 Participants

The target population consisted of Peruvian youths aged 18–30 years, with an estimated national projection of 7,867,000 individuals (Instituto Nacional de Estadística e Informática, 2023). Participants were selected through stratified probabilistic sampling by natural geographic region (coast, highlands, and rainforest), following the INEI classification. Based on the formula for finite populations, a minimum sample size of 384 participants was estimated; however, the final sample comprised 967 Peruvian youths (M = 23.57, SD = 3.69; females = 577, males = 390), increasing the statistical power of the study. Inclusion criteria were Peruvian nationality, enrollment in secondary, university, or technical education, provision of informed consent, and no fluent command of English. Individuals with dual nationality, cognitive disabilities, or English as their mother tongue were excluded.

### 2.3 Instrument

The Foreign Language Learning Enjoyment Scale in the Classroom (EDALEC) was used as the assessment instrument in this study. The original version of the scale was developed in English by Dewaele and MacIntyre (2014) and consisted of 21 items designed to assess the emotional dimensions associated with enjoyment in foreign language learning. The original scale demonstrated adequate internal consistency (*α* = 0.86).

Subsequently, Acosta-Manzano and Barrios (2022) conducted the translation, cultural adaptation, and psychometric validation of a short Spanish version of the scale composed of nine items. This version was organized into three dimensions based on the proposal of Botes et al. (2021): Teacher-induced enjoyment (items 1–3; *α* = 0.85), Personal enjoyment (items 4–6; *α* = 0.66), and Social enjoyment (items 7–9; *α* = 0.80). Each dimension was assessed using a five-point Likert-type scale, where 1 indicated strongly disagree and 5 indicated strongly agree.

Regarding its psychometric properties, the validation conducted by Acosta-Manzano and Barrios (2022) supported a stable hierarchical structure, adequate construct validity through confirmatory factor analysis, and acceptable reliability levels across the three dimensions.

### 2.4 Procedure

Prior to data collection, formal authorization was obtained from the original authors of the instrument through written communication, in accordance with the ethical guidelines established by the ITC (International Test Commission, 2017) for the use and adaptation of psychometric tests. Data collection was conducted between March and June using a mixed modality, combining face-to-face administration in selected educational institutions and online administration via the Google Forms platform. This approach facilitated access to participants from different regions of the country, including the coast, highlands, and jungle.

During data collection, participation was voluntary, anonymous, and confidential. In addition, informed consent was obtained from all participants in accordance with the ethical principles of the Declaration of Helsinki (World Medical Association, 2013) and with prior approval from the Ethics Committee of the Universidad Peruana Unión. Subsequently, the responses were coded and cleaned for analysis. Exploratory and confirmatory factor analyses were conducted to examine the factorial structure of the instrument, assess measurement invariance by sex, and estimate internal consistency indices.

### 2.5 Data analysis

Several statistical analyses were conducted to examine the psychometric properties of the EDALEC. First, descriptive statistics were calculated for each item, including means, standard deviations, skewness, kurtosis, and corrected item-total correlations. Skewness and kurtosis values within ±1.5 were considered acceptable for evaluating the distribution of the items (Alatlı, 2020; Kline, 2023; Pérez and Medrano, 2010).

To strengthen the validation process and reduce the risk of overfitting, the total sample (*N* = 967) was randomly divided into two independent subsamples. The first subsample (*n* = 349) was used for the exploratory factor analysis (EFA), whereas the second subsample (*n* = 618) was used for the confirmatory factor analysis (CFA) and measurement invariance testing by sex. This procedure allowed the factor structure identified in the exploratory phase to be tested in an independent confirmatory subsample (Worthington & Whittaker, 2006; Brown, 2015).

Since the items were answered on a five-point Likert-type scale, the EFA was conducted using a polychoric correlation matrix which is appropriate for ordinal indicators (Bandalos and Finney, 2018). Sampling adequacy was assessed through the Kaiser–Meyer–Olkin (KMO) index and Bartlett’s test of sphericity Kaiser (1974). The number of factors was determined using parallel analysis and inspection of the scree plot. Factors were extracted using the minimum residuals (MINRES) method and rotated with oblimin rotation, as correlations among the dimensions of foreign language enjoyment were expected (Bernaards and Jennrich, 2005; Lloret-Segura et al., 2014; Tabachnick and Fidell, 2019).

The CFA was then performed to test the three-factor model proposed by Botes et al. (2021), which includes Teacher-induced enjoyment, Personal enjoyment, and Social enjoyment. Because the items were ordinal, the model was estimated using robust weighted least squares with mean and variance adjustment (WLSMV), an estimator recommended for ordinal indicators (Flora and Curran, 2004; DiStefano and Morgan, 2014). Model fit was evaluated using CFI, TLI, RMSEA, and SRMR. CFI and TLI values ≥0.90 were considered acceptable, and values ≥0.95 were considered excellent. RMSEA and SRMR values ≤0.08 were interpreted as acceptable indicators of fit (Hu and Bentler, 1999; Kline, 2023).

Measurement invariance by sex was evaluated through configural, metric, scalar, and strict models.

Configural invariance examined whether the same factor structure was present in males and females. Metric invariance tested the equivalence of factor loadings, scalar invariance tested the equivalence of item thresholds, and strict invariance tested the equivalence of residual variances. Invariance was evaluated using changes in fit indices, with ΔCFI ≤ 0.010 and ΔRMSEA ≤ 0.015 as criteria for acceptable invariance (Chen, 2007; Putnick and Bornstein, 2016).

The relationships among the latent dimensions were examined through interfactor correlations obtained in the CFA. These correlations were interpreted to describe the degree of association among Teacher-induced enjoyment, Personal enjoyment, and Social enjoyment, without assuming that the dimensions were completely independent.

Finally, internal consistency was estimated using ordinal alpha and McDonald’s omega, as both coefficients are appropriate for ordinal items and congeneric measurement models (Gadermann et al., 2012; Dunn et al., 2014). All analyses were performed in R version 4.1.1. The EFA was conducted using the psych and GPArotation packages, while the CFA and measurement invariance analyses were conducted using lavaan and semTools (Rosseel, 2012; Jorgensen et al., 2021) (see Table 1).

**Table 1:** Sociodemographic information.

Category	Frequency	%
Gender		
Female	577	59.7%
Male	390	40.3%
Region of origin		
Coastal	474	49.0%
Highlands	290	30.0%
Jungle	203	21.0%
Language being learned		
English	922	95.3%
Other	45	4.7%

## 3 Results

### 3.1 Descriptive analysis of the items

Table 2 presents the descriptive statistics for the nine items of the EDALEC. Item means ranged from 3.39 for item D8 to 4.03 for item D2, indicating a general tendency toward high levels of reported foreign language enjoyment. Item D7 showed the highest standard deviation (SD = 1.107), suggesting greater variability in participants’ responses, particularly regarding shared humor experiences in the classroom.

**Table 2:** Descriptive analysis of the Items.

Items	M	DE	*g*₁	*g*₂	ritc
D1	3.85	1.006	-0.801	0.257	0.78
D2	4.03	0.939	-1.052	1.063	0.76
D3	3.88	0.983	-0.851	0.495	0.8
D4	3.81	1.015	-0.684	0.076	0.73
D5	3.94	0.937	-1.011	1.061	0.73
D6	3.78	1.012	-0.743	0.218	0.73
D7	3.46	1.107	-0.432	-0.445	0.58
D8	3.39	1.083	-0.338	-0.412	0.56
D9	3.43	1.097	-0.412	-0.438	0.54

M = mean, SD = standard deviation, *g*₁ = skewness, *g*₂ = kurtosis, ritc = corrected item–total correlation.

Skewness (*g*₁) and kurtosis (*g*₂) values were within the acceptable range of ±1.5 (Pérez & Medrano, 2010), indicating no severe deviations in the distribution of the items. In terms of item discrimination, all corrected item-total correlations (ritc) exceeded the recommended threshold of 0.30, suggesting adequate discriminative capacity across the nine items.

#### 3.1.1 Exploratory factor analysis

The exploratory factor analysis (EFA) was conducted with a randomly selected subsample of 349 participants, obtained after dividing the total sample into two independent subsamples for crossvalidation purposes. This procedure was applied to reduce the risk of overfitting and to allow the factorial structure identified in the exploratory phase to be tested later in an independent confirmatory subsample.

**Figure 1:**
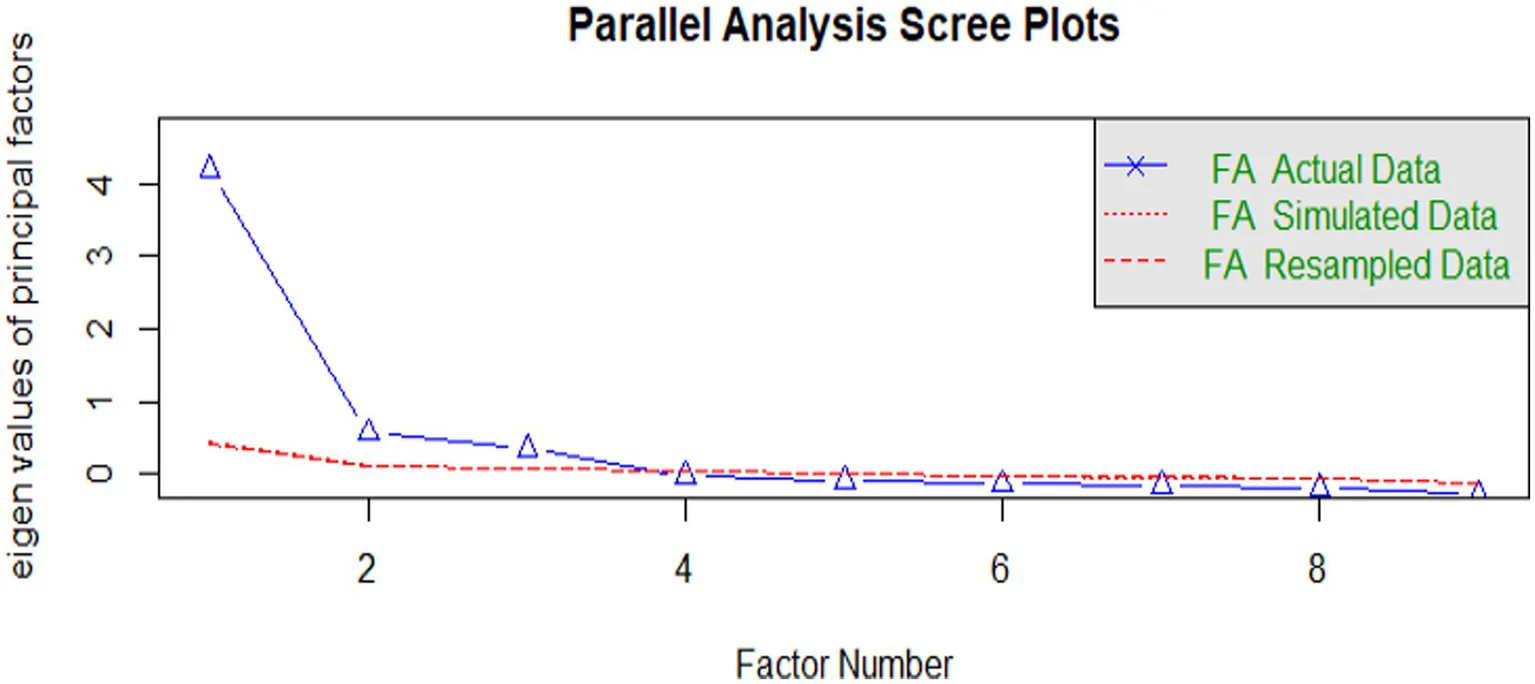
Parallel analysis of the Foreign Language Enjoyment Scale.

The data showed adequate conditions for factor analysis. The Kaiser–Meyer–Olkin index indicated good sampling adequacy (KMO = 0.86), and Bartlett’s test of sphericity was statistically significant, *χ*^2^(36) = 1517.00, *p* < 0.001, indicating that the correlation matrix was suitable for factor extraction. The first four empirical eigenvalues were 4.46, 1.51, 0.77, and 0.56, respectively. Parallel analysis indicated that the first three observed eigenvalues exceeded those obtained from randomly generated data, whereas the fourth eigenvalue did not (Horn, 1965). Therefore, a three-factor solution was retained. The scree plot also showed an inflection after the third factor, supporting the theoretical structure of the short version of the Foreign Language Enjoyment Scale. Factor retention decisions were based on empirical evidence and theoretical interpretability, following recommendations for exploratory factor analysis (Fabrigar and Wegener, 2012) (see Figure 1).

As shown in Table 3, all items presented factor loadings above the recommended minimum value of 0.30. Item D2 showed the highest loading (0.94) on the Teacher-induced enjoyment factor, whereas item D8 showed the lowest loading (0.64) on the Social enjoyment factor. The Teacher-induced enjoyment factor included items D1, D2, and D3, with loadings ranging from 0.63 to 0.94. The Personal enjoyment factor included items D4, D5, and D6, with loadings ranging from 0.68 to 0.88. The Social enjoyment factor included items D7, D8, and D9, with loadings ranging from 0.64 to 0.89.

**Table 3:** Exploratory factor analysis of the Foreign Language Enjoyment scale

Items	Teacher appreciation	Personal enjoyment	Social enjoyment
D2	0.94		
D3	0.79		
D1	0.63		
D5		0.88	
D4		0.78	
D6		0.68	
D9			0.89
D7			0.72
D8			0.64
Explained variance (%)	0.24	0.24	0.2
Cumulative variance (%)	0.24	0.49	0.68
Factores	Teacher appreciation	Personal enjoyment	Social enjoyment
Teacher appreciation	1		
Personal enjoyment	0.73	1	
Social enjoyment	0.39	0.36	1

*h*^2^ = communalities.

Together, the three factors explained 68% of the total variance, with Teacher-induced enjoyment accounting for 24%, Personal enjoyment for 24%, and Social enjoyment for 20%. Factor loadings ranged from 0.63 to 0.94, exceeding the recommended minimum criterion of 0.30. Interfactor correlations ranged from 0.36 to 0.73, indicating moderate to strong positive associations among the three dimensions while supporting their empirical distinctiveness.

#### 3.1.2 Confirmatory factor analysis and measurement invariance by sex

The three-dimensional model was evaluated through confirmatory factor analysis using the second independent subsample of 618 participants. To further evaluate construct validity, the proposed three factor model was compared with alternative factorial structures, including a unidimensional model and a second-order model. The three-factor model demonstrated an excellent fit to the data (*χ*^2^ = 48, df = 24, CFI = 0.999, TLI = 0.998, RMSEA = 0.040, SRMR = 0.008), whereas the unidimensional model (*χ*^2^ = 494, df = 27, CFI = 0.999, TLI = 0.998, RMSEA = 0.040, SRMR = 0.031) and the second-order model (*χ*^2^ = 48, df = 24, CFI = 0.999, TLI = 0.998, RMSEA = 0.040, SRMR = 0.031) also showed acceptable fit. However, the three-factor model was retained because it was theoretically consistent with the conceptualization of foreign language enjoyment and provided the best overall fit, particularly as indicated by the lowest SRMR. Convergent validity was supported by Average Variance Extracted (AVE) values of 0.804 for Teacher-induced enjoyment, 0.666 for Personal enjoyment, and 0.549 for Social enjoyment, all exceeding the recommended threshold of 0.50.

Discriminant validity was assessed using the heterotrait–monotrait ratio (HTMT), yielding values of 0.708, 0.665, and 0.759, all below the recommended cutoff of 0.85, indicating that the three factors represent related but distinct constructs. Finally, although the correlation between Teacher-induced enjoyment and Social enjoyment increased from 0.665 in the exploratory factor analysis to 0.78 in the confirmatory factor analysis, this difference is expected because the EFA and CFA were conducted on independent random subsamples and estimated interfactor relationships using different statistical approaches. Therefore, these differences likely reflect normal sampling variability rather than inadequate discriminant validity.

**Figure 2:**
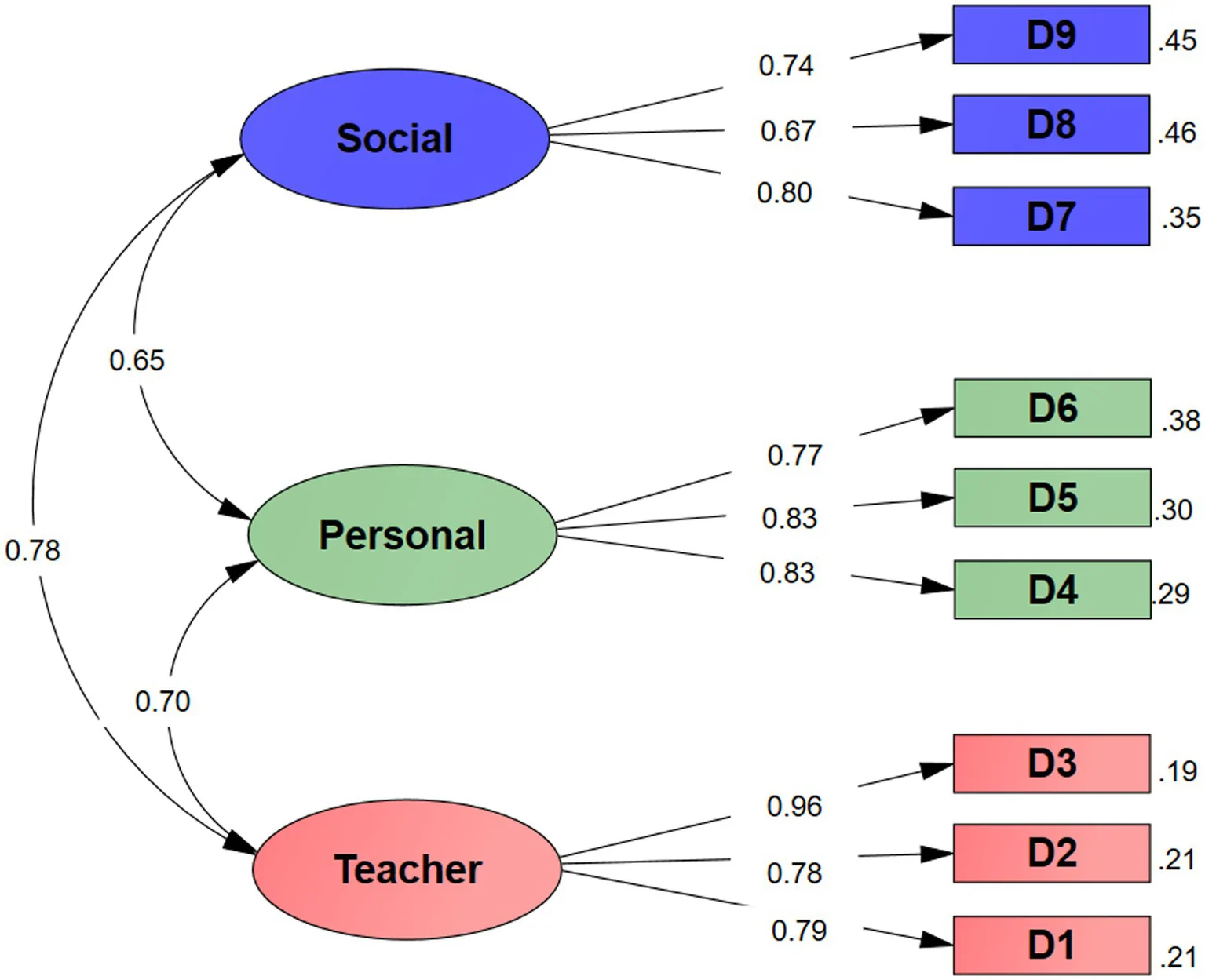
Internal structure of the Foreign Language Enjoyment Scale.

As shown in Figure 2, all standardized factor loadings were above 0.50, indicating that the items were adequately associated with their respective dimensions. The Teacher-induced enjoyment dimension showed loadings ranging from 0.78 to 0.96, the Personal enjoyment dimension from 0.77 to 0.83, and the Social enjoyment dimension from 0.67 to 0.80.

The correlations among the latent factors were positive and ranged from moderate to high. Teacher-induced enjoyment was associated with Personal enjoyment (*r* = 0.70) and Social enjoyment (*r* = 0.78), while Personal enjoyment was associated with Social enjoyment (*r* = 0.65). These results suggest that the three dimensions are closely connected components of foreign language enjoyment while still representing conceptually distinct aspects of the construct.

Measurement invariance by sex was examined to determine whether the EDALEC measured foreign language enjoyment equivalently in males and females. As reported in Table 4, the configural model showed CFI = 0.998 and RMSEA = 0.025; the metric model showed CFI = 0.997 and RMSEA = 0.022; the scalar model showed CFI = 0.996 and RMSEA = 0.021; and the strict model showed CFI = 0.996 and RMSEA = 0.024. Across the sequence of models, the changes in CFI and RMSEA were minimal, supporting measurement invariance by sex according to the criteria used in the study.

**Table 4:** Measurement invariance analysis by sex.

Model	*χ* ^2^	Δ*χ*^2^	df	Δdf	*p*	CFI	ΔCFI	**TLI**	**ΔTLI**	**SRMR**	**ΔSRMR**	RMSEA	ΔRMSEA
Configural	10.564		12		—	0.998		0.996		0.027		0.025	—
Metric	14.624	–4.06	16	–4	0.016	0.997	0.001	0.996	—	0.03	–0.003	0.022	0.003
Scalar	16.448	–1.824	20	–4	0.017	0.996	0.001	0.997	–0.001	0.031	–0.001	0.021	0.001
Strict	28.135	–11.687	26	–6	0.004	0.996	0	0.996	0.001	0.036	–0.005	0.024	−0.003

Δ*χ*^2^ = change in chi-square; Δdf = change in degrees of freedom; *p* = probability value; ΔCFI = change in comparative fit index; ΔRMSEA = change in root mean square error of approximation.

#### 3.1.3 Internal consistency

The reliability of the three dimensions was evaluated using ordinal alpha and McDonald’s omega coefficients. The Teacher-induced enjoyment dimension showed excellent reliability (*α* = 0.92; *ω* = 0.88). The Personal enjoyment dimension showed adequate reliability (*α* = 0.85; *ω* = 0.81), and the Social enjoyment dimension also showed adequate reliability (*α* = 0.77; *ω* = 0.75). These values indicate acceptable to excellent internal consistency across the three dimensions of the EDALEC.

## 4 Discussion

The present study examined the psychometric properties of the short version of the Foreign Language Learning Enjoyment Scale in the Classroom (EDALEC) in a sample of Peruvian youth. This study responds to the need for brief and psychometrically supported instruments that can assess emotional experiences in foreign language learning within Spanish-speaking contexts, which remain underrepresented in the international psychometric literature.

The results of the exploratory and confirmatory factor analyses supported a three-dimensional structure composed of Teacher-induced enjoyment, Personal enjoyment, and Social enjoyment. To strengthen the evaluation of this factorial structure and reduce the risk of overfitting, the total sample was divided into two independent subsamples: one for the exploratory factor analysis and another for the confirmatory factor analysis. This procedure provides greater confidence in the stability of the proposed structure (van Prooijen and van der Kloot, 2001; Fokkema and Greiff, 2017; Lorenzo-Seva, 2022). Overall, the findings suggest that enjoyment in foreign language learning should not be understood as a single, undifferentiated experience, but as a multidimensional construct shaped by personal, pedagogical, and social factors. This interpretation is consistent with positive psychology approaches applied to language learning (Dewaele and MacIntyre, 2014; Wang and Jiang, 2022).

From a theoretical perspective, the results highlight the importance of the classroom environment in students’ emotional experiences. Enjoyment does not appear to depend only on students’ individual characteristics, but also on the quality of teacher support and peer interaction. In particular, the Teacher-induced enjoyment dimension reinforces the central role of the teacher in creating a positive emotional climate. Supportive, kind, and motivating teaching practices may help students experience foreign language learning as more enjoyable and meaningful (Dewaele and MacIntyre, 2014; Jin and Zhang, 2019; Li et al., 2018).

Regarding reliability, the ordinal alpha and omega coefficients showed adequate internal consistency across the three dimensions. These findings are consistent with previous validations of short versions of Foreign Language Enjoyment Scales in other contexts. They also suggest that reducing the number of items does not necessarily weaken the quality of measurement, as long as the conceptual coherence of the instrument is preserved.

The confirmatory findings reinforce the multidimensional nature of the construct proposed in this study. The overall pattern of evidence supports the adequacy of the measurement model, indicating that the latent dimensions are empirically distinguishable while remaining theoretically related. This interpretation is consistent with contemporary recommendations that emphasize evaluating construct validity through multiple sources of evidence, including model fit, convergent validity, and discriminant validity, rather than relying on a single statistical indicator (Kline, 2023; Hair et al., 2022; Henseler et al., 2015). Consequently, the results provide robust support for the factorial validity of the instrument and its application in the assessment of enjoyment among university students.

When compared with previous studies, the findings are consistent with the short version proposed by Botes et al. (2021), which also identified components related to the teacher, Personal enjoyment, and social interaction. They are also in line with the Spanish adaptation by Acosta-Manzano and Barrios (2022), although that study was conducted with a smaller European sample. In this sense, the present study contributes evidence of validity based on internal structure and measurement invariance in a Latin American youth population, expanding the study of foreign language enjoyment in Spanish-speaking educational contexts.

Another relevant contribution is the evidence of measurement invariance by sex at the configural, metric, scalar, and strict levels. This finding suggests that the EDALEC measures foreign language enjoyment in a comparable way among males and females. Therefore, comparisons between these groups can be interpreted with greater confidence, as differences are less likely to be due to measurement bias.

Despite these strengths, some limitations should be acknowledged. First, although the short version of the EDALEC showed favorable evidence based on internal structure, reliability, and measurement invariance by sex, its use in other educational and cultural contexts requires further psychometric evaluation. This is particularly relevant given the linguistic, regional, and sociocultural diversity of Latin American countries. Second, the study relied on a self-report measure; therefore, participants’ responses may have been influenced by social desirability, subjective interpretation of the items, or response styles. Third, because of the cross-sectional design, it was not possible to examine the temporal stability of foreign language enjoyment or potential changes in this construct over time. Fourth, although the sample was large and included Peruvian youth from different geographic regions, most participants were learning English, which limits the generalizability of the findings to other foreign language learning contexts. Finally, although measurement invariance by sex was supported, future studies should examine whether the scale operates equivalently across geographic regions, educational levels, age groups, and other sociocultural contexts. In addition, future research should incorporate external sources of validity evidence, including convergent, divergent, and criterion-related validity.

In conclusion, the findings provide evidence of validity based on internal structure and adequate internal consistency for the short version of the EDALEC in Peruvian youth. The exploratory and confirmatory factor analyses supported a three-dimensional structure composed of Teacher-induced enjoyment, Personal enjoyment, and Social enjoyment. In addition, measurement invariance by sex was supported at the configural, metric, scalar, and strict levels, suggesting that the scale operates equivalently in males and females. These results support the use of the short version of the EDALEC as a brief and psychometrically supported instrument for assessing foreign language enjoyment in Latin American educational contexts. However, further studies are needed to examine its performance in other regions, educational levels, age groups, and foreign language learning contexts.

## Data Availability

The datasets generated and/or analyzed during the current study are available from the corresponding author upon reasonable request.
